# Soft tissue prediction in orthognathic surgery: Improving accuracy by means of anatomical details

**DOI:** 10.1371/journal.pone.0294640

**Published:** 2023-11-27

**Authors:** Federica Ruggiero, Alessandro Borghi, Mirko Bevini, Giovanni Badiali, Ottavia Lunari, David Dunaway, Claudio Marchetti

**Affiliations:** 1 DIBINEM, Alma Mater Studiorum University of Bologna, Bologna, Italy; 2 Department of Engineering, Durham University, Durham, United Kingdom; 3 Oral and Maxillofacial Surgery Unit, IRCCS AOU di Bologna, Bologna, Italy; 4 Craniofacial Unit, Great Ormond Street Hospital, London, United Kingdom; Justus Liebig University Giessen, GERMANY

## Abstract

Three-dimensional virtual simulation of orthognathic surgery is now a well-established method in maxillo-facial surgery. The commercial software packages are still burdened by a consistent imprecision on soft tissue predictions. In this study, the authors produced an anatomically detailed patient specific numerical model for simulation of soft tissue changes in orthognathic surgery. Eight patients were prospectively enrolled. Each patient underwent CBCT and planar x-rays prior to surgery and in addition received an MRI scan. Postoperative soft-tissue change was simulated using Finite Element Modeling (FEM) relying on a patient-specific 3D models generated combining data from preoperative CBCT (hard tissue) scans and MRI scans (muscles and skin). An initial simulation was performed assuming that all the muscles and the other soft tissue had the same material properties (Homogeneous Model). This model was compared with the postoperative CBCT 3D simulation for validation purpose. Design of experiments (DoE) was used to assess the effect of the presence of the muscles considered and of their variation in stiffness. The effect of single muscles was evaluated in specific areas of the midface. The quantitative distance error between the homogeneous model and actual patient surfaces for the midface area was 0.55 mm, standard deviation 2.9 mm. In our experience, including muscles in the numerical simulation of orthognathic surgery, brought an improvement in the quality of the simulation obtained.

## Introduction

Orthognathic surgery is a well-established practice aimed to restore both morphology and function in congenital or acquired jaw deformities by means of several types of osteotomies. Successful surgery depends on surgical expertise and accurate pre-surgical planning.

Accurate surgical planning requires a precise simulation of osteotomies, bone segment movements as well as the precise prediction of facial soft tissue changes induced by bony movements [1–3]. Due to the complex physical properties of facial soft tissues, accurate post-surgical predications are challenging even with accurate osteotomy simulations [1–5]. Furthermore, it is well recognized in the Literature that the middle third of the face (i.e. nose, cheeks, upper lip) is the area which proves most difficult to accurately predict as it doesn’t respond in a proportional fashion according to bone movement after LeFort osteotomies [1–3].

The most commonly adopted techniques for simulation of soft tissue changes are the finite element modeling (or finite element method ‐ FEM), mass-spring model (MSM), and mass tensor model (MTM) [4]. Comparison of these three techniques reported in the literature shows that FEM allows for better simulation of hard and soft tissue biomechanical interaction which leads to more accurate results [1–3, 5, 6]. FEM was first developed in 1940s for use in aeronautical engineering ad became popular for surgical simulation since 1990s. It is a mathematical modelling technique whereby a complex object (such as a patient face) is subdivided into smaller elements (such as bony elements and soft tissue elements) whose mechanical response to external loads (such as orthognathic segment repositioning) can be retrieved by solving continuum mechanics equations analytically by means of a system of algebraic equations. The overall body response is then calculated by combining the response of each element while taking into account the relative position and the mutual interactions [7–9].

The accuracy of FEM simulation depends on the geometrical fidelity of the FE mesh, on the accuracy of the constitutive tissue mechanical properties and on the adoption of realistic boundary conditions [7]. Commercial software (such as Proplan, Dolphin, Simplant O&O, Maxilim) [10] are accurate in reproducing the behavior of hard tissues, whereas prediction of soft tissue proves challenging due to the difference in elasticity of the several components of the face (muscles, mucosa, fascia, etc.). To date, commercially available surgical planning software cannot reliably predict soft tissue outcome [10–14].

In the years several efforts have been made to improve the models and to incorporate more anatomical details with different biomechanical properties [11–17]. MRI scans provide the best definition to facial muscles when segmented from DICOMs [18]. Previous works on facial tissue characterization have demonstrated that different areas of facial soft tissues have different biomechanical properties in terms of longitudinal tissue stiffness (Young’s modulus- E) and transverse behavior (Poisson’s ratio - ν) [19].

In this study, the Authors built on their previous efforts in producing accurate patient specific 3D mid-face models to predict the outcome of orthognathic surgery [20, 21] and assess the effect of surgical parameters on the aesthetic outcome [22]; the validated modelling technique was enhanced by combining different imaging modalities (CBCT, MRI) and create patient specific anatomically detailed 3D models of the face and facial muscles, to test the hypothesis that the addition of muscles–having different material properties compared to the rest of the soft tissues ‐ had an effect on the reshaping prediction outcome of orthognathic surgery. An attempt to optimize FEM material properties in view of a probabilistic prediction of soft tissue outcomes has been previously published by our group [20]. In the current work, our focus was to validate an accurate model which comprehends mimic and masticatory muscles, focusing particularly on the midface area and its changes after the LeFort I advancement.

## Materials and methods

### Patient population and surgery

Eight consecutive patients admitted at St Orsola–Malpighi University Hospital (Alma Mater Bologna), Oral and Maxillo-facial Unit and undergoing bimaxillary orthognathic surgery were included in this study. Written informed consent was obtained from all subjects involved in the Study; the Study obtained the approval of the local Ethical Committee (protocol number 1015/2021/Oss/AOUBo ). Throughout the duration of the study (12 months), we enrolled 5 female and 3 males, mean age of 33.9 +/- 16.5 (range 18–66 years, median 29 years). Our cohort included patients undergoing orthognathic surgery for malocclusion or OSAS and not wearing orthodontic devices (which would pose issues for the MRI scan) prior to surgery, non claustrophobic, aged over 18 who signed the informed consent before the beginning of the study. Patients with high anesthesiology risk, immunosuppressed or immunocompromised, with comorbidities such as diabetes, pregnant women, needing presurgical orthodontics and with extensive facial scarring due to previous trauma and/or intervention, were excluded. Bimaxillary surgery was performed by a single surgeon (GB). Presurgical virtual planning was performed according to the standard protocol using a preoperative planning software (Simplant, Materialise, Leuvenn, Belgium). Surgical wafers were produced as usual to allow the jaws repositioning.

### Imaging acquisition

All the patients underwent standard presurgical evaluation by means of planar x-rays and CBCT scans (which is the standard for orthognathic patients). All CBCT scans were obtained from the same machine NewTom 3000 VGI Evo (Cefla Group, Imola, Italy) with the patient in natural head position and standing. Patient occlusion was ensured by asking them to bite a centric occlusion wax cast. After postoperative discharge, all the patients underwent postoperative CBCT scan (using the same machine as the preoperative scan). Only five postoperative CBCT scans were suitable for validation (i.e. six months after surgery, when the postoperative edema was resolved).

In addition, each patient received a preoperative MRI with the same protocol (3D DWI sequence on GE 1.5 Tesla machine), with the patient supine in the gantry.

### Data processing and creation of the FE model

**Fig 1:**
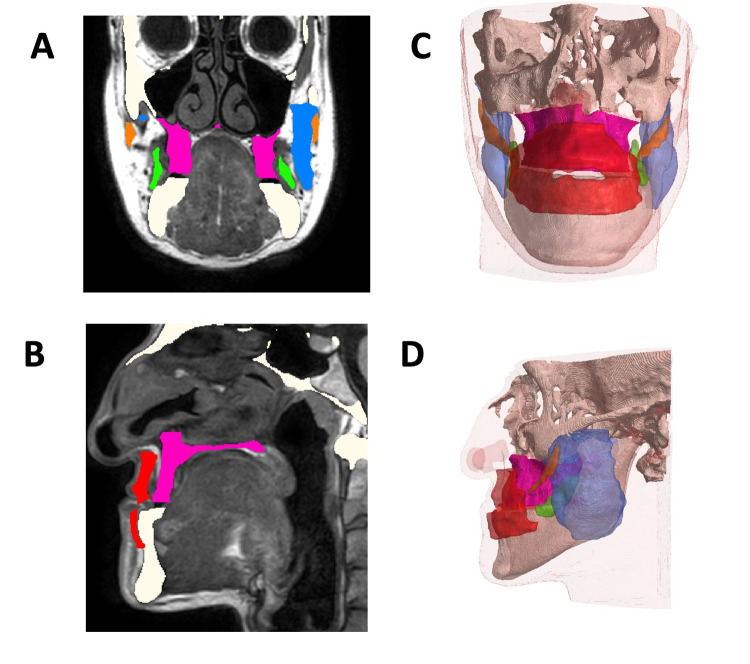
Construction of the model A: axial view on MRI with highlighted segmented muscles in different colors B: sagittal view on CBCT scan whereas bone has been segmented C frontal and D latera view of the merged 3D model obtained with CBCT 3D bone and MRI 3D muscles.

All DICOM data were extracted both from preoperative CBCT and MRI scans and from postoperative CBCT scans. The datasets were separately imported in Simpleware ScanIP software (Synopsis, USA), where each component (hard tissue from CBCT, and soft tissue and muscles from MRI) was rendered three-dimensionally. The skull and mandible were extracted from the preoperative CBCT (white in Fig 1A and 1B); the LeFort I fragment was separated from the skull base using postoperative planar x-ray and CBCT as reference (pink, in Fig 1A and 1B). The following muscles were segmented from the preoperative MRI: masseter (M, left and right, blue in Fig 1A), buccinator (B, left and right, green in Fig 1A), orbicularis oris (O, red in Fig 1B), zygomaticus major (Z, left and right, orange in Fig 1A). The muscles considered have been segmented by manual thresholding and by comparing the anatomic structure with the virtual atlas VISU [23]. Minor mimic muscles (i.e. risorius, levator labii) were segmented as one block with the skin and other subcutaneous tissues. Landmark based registration was performed to position the segmented skull (and LF1 segment) according to the position of the muscles and other soft tissues on the MRI dataset to produce a combined hard and soft tissue dataset model (Fig 1A and 1B). Minor adjustments to the 3D volumes (localized smoothing, local void filling) were performed to ensure a good quality 3D mesh models could be produced (Fig 1C and 1D) and to remove unnecessary details which would impact the mesh size without affecting the simulation results. Each mesh was created using the same set of discretization parameters and it was imported in ANSYS Workbench 2020 (Canonsburg, PA, USA).

### FE simulation of orthognathic surgery, validation

**Fig 2:**
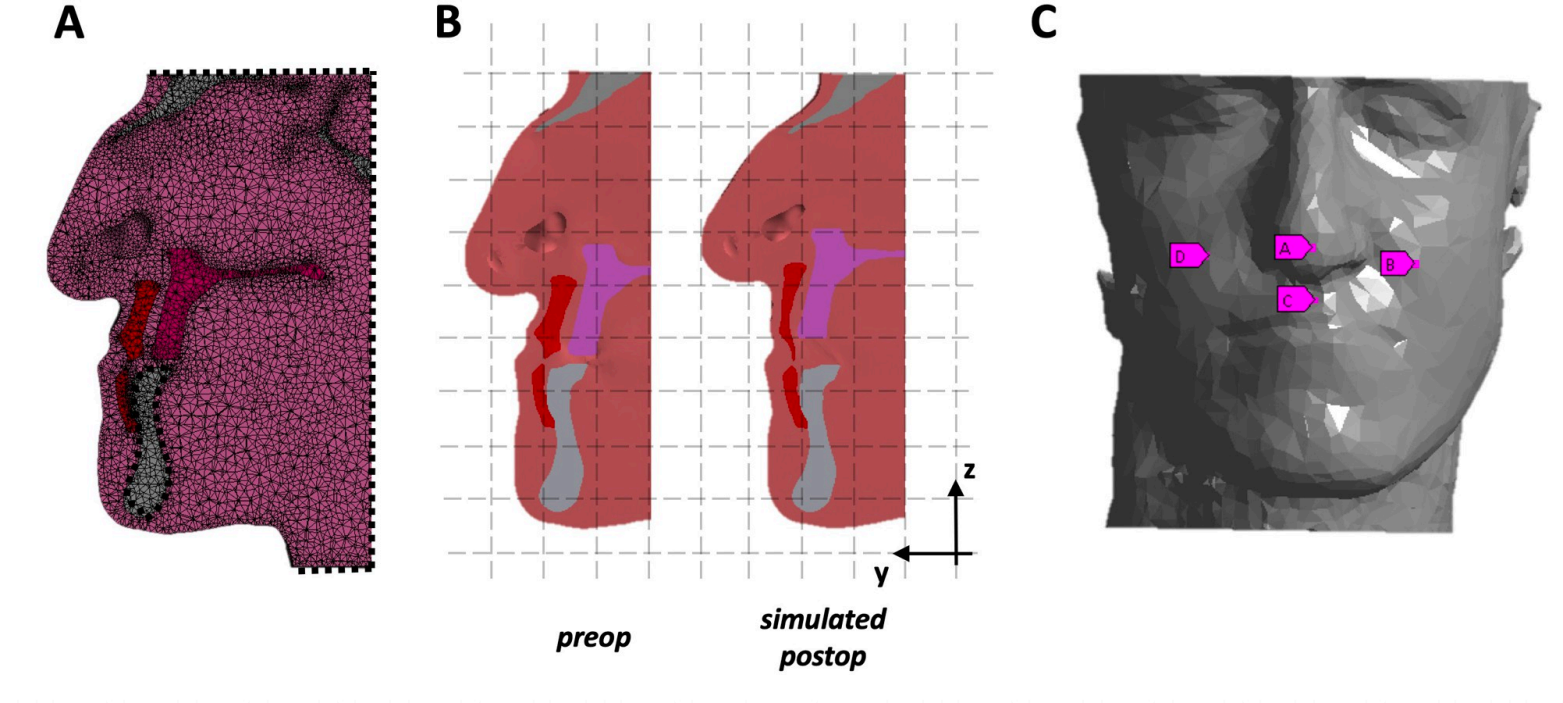
FEM on patient’s model A: model in FE, with constraints applied B: simulation of the bone displacement C: 4 landmarks selected as output in FE for the DoE.

In Ansys Workbench, boundary conditions were applied to mimick the tethering of the other tissues (top of the head, back of the head, neck) on the FE model: each surface was fully constrained in terms of rotation and translation (Fig 2A). The mandible was also fully constrained. The orthognathic repositioning was simulated by applying a patient-specific displacement on the LF1 segment along the Y and Z axis (Fig 2B), which replicated the horizontal advancement (5.4mm ± 3.2mm) and vertical impaction (3.8mm±1.6mm) performed during surgery.

The FE software allows to attribute to each component of the model specific biomechanical properties established from the Literature [20]. For each simulation, the soft tissues were assumed to have E = 0.1MPa and ν = 0.45; the hard tissues (skull base, LF1 segment and mandible) were assumed to have a Young’s modulus equal to 5GPa and a Poisson’s ratio equal to 0.2.

A first simulation was performed for each patient where all muscles were assumed to have the same material properties as the other soft tissues (Table 1 - homogeneous model H). Such model was used for validation and for base reference.

**Table 1:** Single muscle material properties in the homogeneous model (H), and min and max values in the design of experiment (DoE).

Muscle	Homogeneous (H) Model	Design of Experiments (DoE)		Reference
	E [MPa]	Min E [MPa]	Max E [MPa]	
Buccinator	0.1	6.7x10^-3^	14.1x10^-3^	24
Masseter	0.1	6.7x10^-3^	14.1x10^-3^	24
Orbicularis Oris	0.1	14.6x10^-3^	22.0x10^-3^	25
Zygomaticus Major	0.1	6.7x10^-3^	14.1x10^-3^	26

The predicted H model was compared against a soft tissue segmentation of a postoperative CBCT in five cases. The analysis was carried out in CloudCompare (Telecom ParisTech/EDR, France) software by means of an iterative closest point alignment of the predicted and postoperative model followed by visual inspection and manual refinement of the alignment if needed. The predicted model was sliced in order to remove surface sections unaffected by the displacement. Once correct alignment was achieved, surface distance (i.e. prediction error) between the surfaces was calculated and visualised by means of colormaps. Average prediction error was calculated for each patient.

### Design of experiment (DOE)

To assess the effect of the presence of the muscles as well as of the physiological variation in muscle stiffness, a design of experiments (DoE [24]) approach was used: a total of 25 simulation were performed, where each muscle stiffness was varied in the prescribed range (Table 1, DoE [23, 25, 26]). DoE is an engineering method used to minimize the number of experiments necessary to characterize parametric sensitivity of a mechanical system. Each parameter combination constitutes a “design point” and the number of design points is calculated by the software according to the DoE type and the number of input parameters (in this case, the four muscle material properties). Optimal Space filling DoE method was adopted as this method maximises the distance between DPs to achieve a more uniform distribution across the design space.

In order to evaluate the system’s behavior on several facial areas, the geometry of the skin surface was divided into four areas: nose (M), upper lip (UL), left and right cheek (LC, RC). These areas were selected as reference landmarks: the total displacement of the four nodes (N, UL, LC, RC, Fig 2C) were employed as output for the design of experiments: The choice of landmarks was bases on literature findings [20, 27]. Local sensitivity was calculated: the sensitivity of each output variables (landmark displacement) to the variation in input (muscle stiffnes) ‐ defined as the rate of output change versus change in input when all other input parameters are maintained constant at the mid-range value ‐ was extracted from the results and compared throughout the population. A positive/negative sensitivity value means that the selected landmark’s displacement increases/decreases with the increase in stiffness of muscle. For simplicity, the sensitivity of left and right cheek (LC, RC) were averaged (C).

To assess statistical difference between the sensitivity of the different node displacement based on muscle stiffness, the Wilcoxon signed-rank test was applied. A p value less than 0.05 was assumed statistically significant. A one-sample Wilcoxon rank test was performed to assess whether individual node sensitivity was statistically different from 0 (i.e. to check if a specific muscle had a consistent effect on a landmark displacement).

From the DoE results, maximum (N_max_, UL_max_, C_max_) and minimum (N_min_, UL_min_, C_min_) displacement for each node for each patient were extracted and compared with the values retrieved from the respective H model results (N_H_, UL_H_, C_H_).

## Results

### Simulation of orthognathic surgery ‐ validation

**Fig 3:**
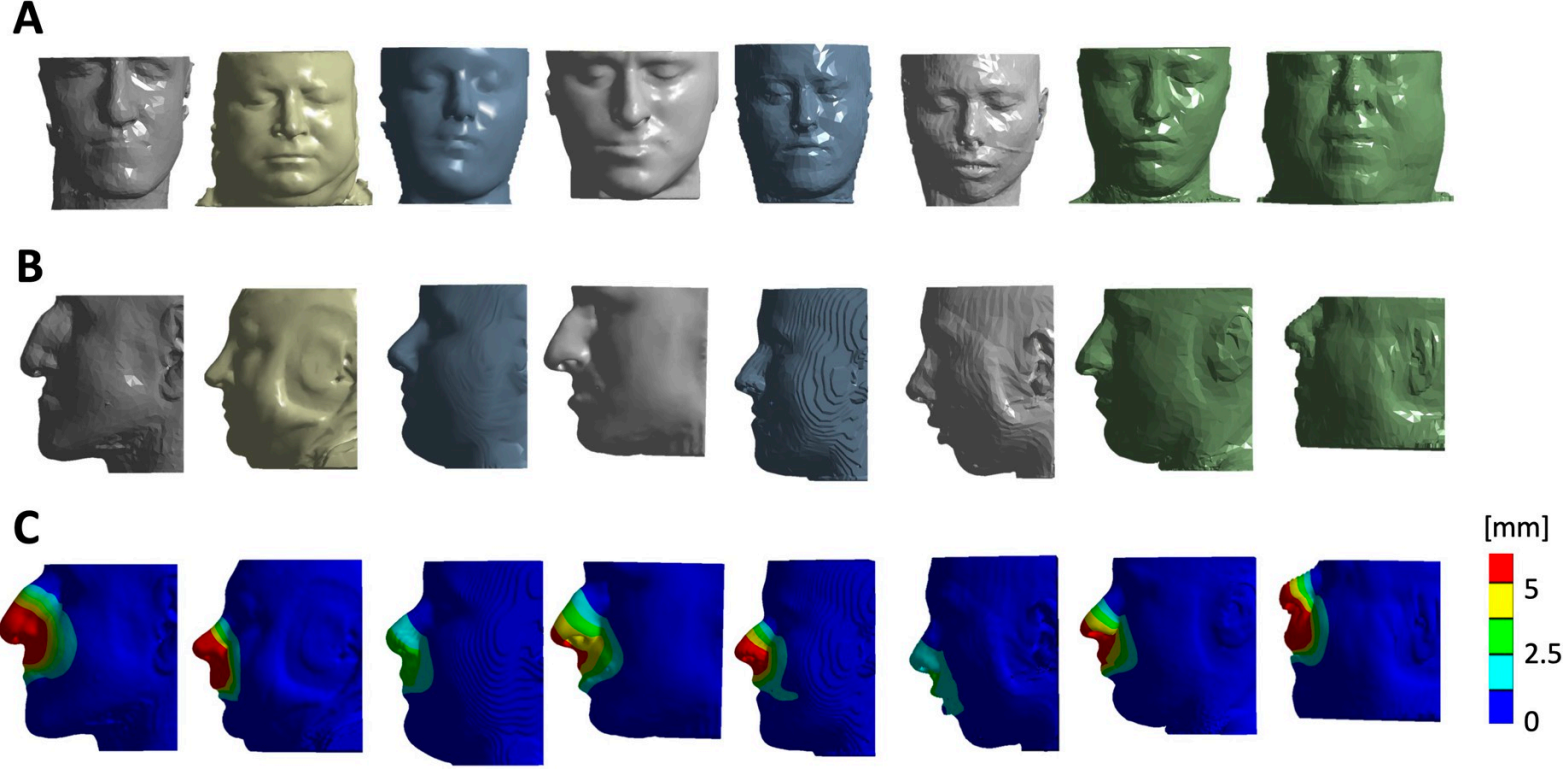
3D model of the patients as imported in Ansys A: frontal view B: lateral view C: graphical visualization of the soft tissue displacement after LeFort 1 simulation.

**Fig 4:**
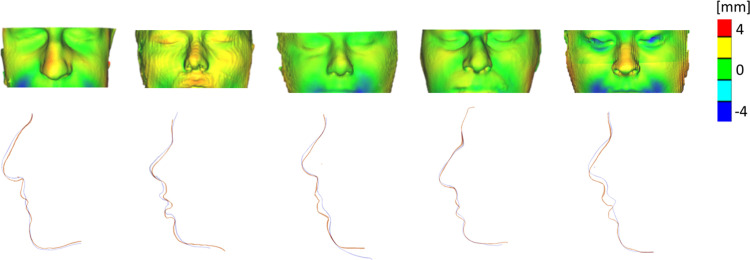
Comparison by means of colormap (top) and sagittal mid-line cross section (bottom) of postoperative 3D soft tissue reconstruction with H model soft tissue reconstruction.

Orthognathic repositioning of the maxilla was simulated by means of FE in 8 patients. Fig 3 shows the surface model reconstructions of the 8 patients as imported in ANSYS (front view in Fig 3A, side view in the Fig 3B); the simulated advancement for the H model of each subject, in terms of total soft tissue displacement, is reported in Fig 3C. Fig 4 shows the comparison between H model surface prediction of 5 subjects with the 3D surface model extracted from postoperative CBCT. Point-to-point distance was extracted and averaged for each patient: the mean overall distance was 0.55mm with an average standard deviation of 2.29mm.

### Muscle sensitivity analysis

Table 2 shows a summary of the sensitivities of each muscle on each node. The last column reports the p-value of the one-way Wilcoxon Signed Rank, to test the null hypothesis that specific muscle sensitivity come from a distribution whose median is zero, which means that the effect of the muscle is not consistent throughout the population. When this hypothesis is rejected (at 5% significance level), it is possible to assume that the variation in muscle stiffness has a consistent effect throughout the population and is not dependent on the anatomy.

**Table 2:** Muscles sensitivities (values are in %).

Landmark		Average (SD)	Range	p-value
N				
	Buccinator	-0.04 (21.09)	[-37.29;32.9]	N.S.
	Masseter	-20.94 (19.93)	[-62.3;-3.61]	<0.05
	Orbicularis Oris	53.13 (43.17)	[-19.32;81.11]	N.S.
	Zygomaticus	-3.72 (5.8)	[-16.02;-0.42]	<0.05
C				
	Buccinator	27.07 (50.56)	[-45.14;84.25]	N.S.
	Masseter	-38.75 (25.92)	[-78.38;-3.79]	<0.05
	Orbicularis Oris	1.23 (10.54)	[-11.2;20.75]	N.S.
	Zygomaticus	-3.29 (3.85)	[-9.68;-0.78]	N.S
UL				
	Buccinator	1.4 (6)	[-7.2;12.18]	1
	Masseter	-5.22 (4.52)	[-13.44;-1.83]	<0.05
	Orbicularis Oris	89.84 (6.89)	[78.48;97.46]	<0.05
	Zygomaticus	-0.36 (1.4)	[-3.51;-0.1]	N.S.

According to the results, the masseter and zygomaticus major had consistently negative effect on the displacement of landmark N (-20.94% +/- 19.93% and -3.72% +/- 5.8% respectively). Relatively to the cheek area, the masseter muscle showed a large spread of sensitivity (from -78.38% to -3.79%) but it always showed a negative sensitivity (-38.75% +/- 25.92%). The UL landmark displacement was negatively influenced by the stiffness of the masseter (-5.22% +/- 4.52%) but was largely positively influenced by the stiffness of the orbicularis oris (89.84% +/- 6.89%).

### Comparison with the homogeneous model

**Fig 5:**
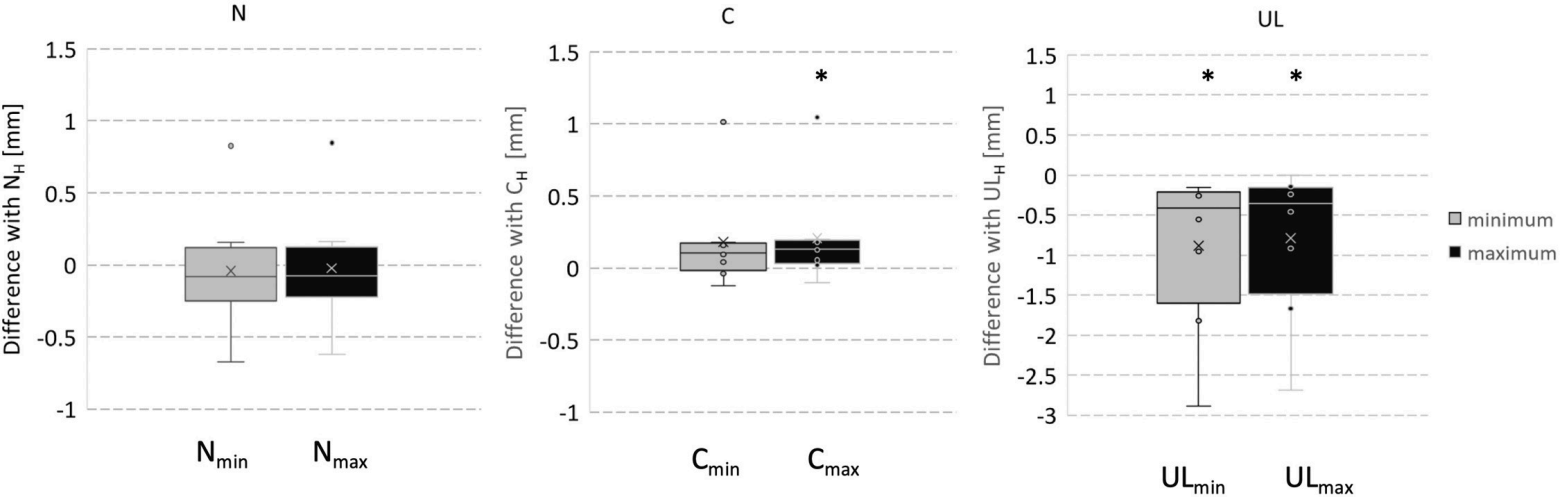
Box-and-Whisker graphs of the distribution of the difference between the maximum (N_max_, UL_max_, C_max_) and minimum (N_min_, UL_min_, C_min_) displacement recorded and the respective H model values.

Fig 5 shows Box-and-Whisker graphs of the distribution of the difference between the maximum (N_max_, UL_max_, C_max_) and minimum (N_min_, UL_min_, C_min_) displacement recorded and the respective H model values (N_H_, UL_H_, C_H_). While for the UL landmark, UL_H_ was consistently higher than UL_max_ (p = 0.012) and UL_min_ (p = 0.012) , for the C landmark the C_H_ displacement values were consistently lower than C_max_ (p<0.05) but that didn’t reach statistical difference with C_min_ (p>0.05). For the N landmark, no clear trend was observed.

## Discussion

Orthognathic surgery aims to reposition patients’ jaws in the most functional and aesthetic way. To achieve this, surgical planning is strongly advisable. Thanks to recent improvements in medical imaging,3D virtual planning provides greater accuracy compared to standard bi-dimensional analysis.

In this work, we have presented a methodology to create an anatomically accurate 3D face model by combining information from preoperative CBCT and MRI images. Precise replication of the osteotomies performed during surgery allowed for retrospective simulation of the maxillary advancement and validation in a subset of patients by comparison with postoperative 3D scan allowed for the quantification of the accuracy of the H model simulation (with encouraging results, compared to other studies in the Literature [12, 13]). Kim et al developed a similar in-house detailed FEM, with a sliding effect of the upper lip, which allowed the group obtain on the upper lip region an accuracy of 1.2mm +/- 0.7mm [11]. These results were promising. Our cohort demonstrated an inaccuracy error of 0.55 mm, with a standard deviation of 2.29.

For the following step, a parametric set of simulations (DoE) was carried out to assess the effect of the inclusion of the muscle (having each a different stiffness value) and to investigate which muscles have the largest effect on the displacement of the different landmarks. The analysis of the sensitivity of the muscle stiffness on the displacement of the landmarks showed that the masseter has a consistently negative effect on the displacement of all the landmarks, i.e. a stiffer muscle would cause a lower displacement on all the points, possibly due to an internal tethering effect. On the other hand, the orbicularis oris muscle stiffness has a consistent positive effect on the displacement of the UL landmark: in this case, a stiffer muscle exhibits lower compression during the orthognathic advancement and transfers the full segment displacement to the upper lip area. No other muscle exhibited consistent behavior throughout the population, except for the zygomaticus major which has a small but consistently negative effect on the N landmark displacement, probably for the same reasons as the masseter.

A further analysis of the results and a comparison of the landmark displacement with the respective H model values showed the effect of introducing muscle stiffness in comparison with assuming a simplified homogeneous behaviour of all the soft tissues (as assumed by standard planning softwares as well as previous works in the literature [10–13, 15]). The results showed that the H model potentially under-predicts the area of the cheek (the H model C displacement values were always lower than the maximum displacement retrieved in the DoE) while it always overestimates the displacement of the UL (both maximum and minimum displacement values were lower–for each patient–than the respective H model values). Similar trends were reported in the Literature relatively to several commercial software [8, 10, 12, 13, 28–30]. Peterman et al reported that Dolphin 3D (Dolphin, Chatsworth, CA, USA) the software was in their experience accurate, but not in the lips area [31]. Dolphin 3D software assumes all soft tissues biomechanically equivalent and therefore may provide a less accurate prediction [31] compared to our models. Bianchi et al. [32] and later Marchetti et al. [33], tested the feasibility in soft tissue predictions of CMF SurgiCase 1.2 software (Materialise, Leuven, Belgium). In both studies the comparison of the prediction versus the actual result was obtained by overlapping 3D surfaces of patients’ faces and using an iterative closest point algorithm as we have done in this study. Their findings showed a percentage error lower than 2 mm but still clinically acceptable, with an increased inaccuracy in the upper lip area [32, 33].

In this study, a modelling framework was provided to produce anatomically accurate face models suitable for performing preoperative planning by means of FEM simulation. The assessment of soft tissue changes was quantified by using facial points which are representative of the areas of the face which are affected by orhognathic surgery, selected in a similar manner to a previous study by our group [20]. Kim et al. [34] analysed soft tissue changes in a group of patients receiving either isolated mandibular surgery or bimaxillary surgery for the treatment of mandibular prognathism by quantifying position changes for a set of soft tissue landmarks. As the movement of different facial regions show correlations in both extent and accuracy [35], it is safe to assume that using a different set of landmarks to assess the output of the DoE would have yielded very similar results. Despite the encouraging results, some limitations of this Study are to mention: a small cohort was included due to the extensive amount of image processing and computational analysis necessary :although 8 patients were recruited to this study, for each patient 25 simulations were carried out to assess sensitivity to single muscle stiffness, making the total number of configurations considered 200. This allowed for parametric analysis and assessment of statistical variation throughout the population. Furthermore, the size of this cohort is in line of that used in several other studies relative to craniofacial and orthognathic surgery planning [20, 21, 32, 33].

Another limitation for this study is the use of different head positions during MRI (supine) and CBCT (neutral) scans, which may affect the relative positions of soft and hard tissues. Although the effect is likely to be minor, as reported in another work by our group where different types of surface scanner outputs were compared, this is yet to be quantified [36].

Furthermore, although the simulation validation was successful, unpredictable postoperative weight loss was noticed which may affect the results. No information on preoperative and postoperative patient weight was recorded, therefore it is difficult to comment how this may have affected change in soft tissue thickness. In this study, BSSO movement was neglected (similarly to Knoops et al. [20]) as this study focused on the movement of the upper jaw.

Limitations of this study are obviously tied to the clinical applicability of the protocol as is: the necessity for a pre-operative MRI limits the applicability to patients who either do not need pre-operative orthodontics or undergo pre-operative orthodontics without fixed appliances.

The operator time needed to segment the muscular structures and the time spent to compute each FEM experiment makes it impractical for use in actual pre-operative planning. It is however possible to use the results of these experiments, after an expansion of the study population, to extract an approximation of the muscular structures from CT scans, to possibly integrate in the pre-operative planning in clinical practice, along with the use of Machine Learning models, as already attempted by other researchers [37–40], to create a mixed deterministic and probabilistic prediction.

A further limitation is caused by the biomechanical properties of the cartilaginous structures of the nose, which were not specifically addressed in the FEM experiments.

## Conclusion

In our experience, relying on this data, it is feasible and recommendable to include different facial anatomical regions (such as the different muscles) in the numerical modelling for the preoperative planning of orthognathic surgery. Particular attention should be paid to the inclusion of the orbicularis oris and masseter muscles, which showed a consistent effect on the landmarks considered and in particular to the upper lip area. Further expansion of the study population could prove useful to refine the results obtained and to move towards clinical applicability.
